# Exiting the ground state: the broad spectrum of cell fates accessible from naïve human pluripotent stem cells

**DOI:** 10.1007/s00018-025-05984-3

**Published:** 2026-01-06

**Authors:** Kyoung-mi Park, Richard Yin, Thorold W. Theunissen

**Affiliations:** Department of Developmental Biology and Center of Regenerative Medicine, Washington University School of Medicine, St. Louis, MO 63110 USA

**Keywords:** Naïve pluripotency, Pluripotent stem cells, Epiblast, Capacitation, Trophectoderm, Trophoblast, Hypoblast, Amnion, Extraembryonic mesoderm, hPGCLCs, 8-cell-like cells, Blastoids

## Abstract

Naïve human pluripotent stem cells (hPSCs) represent an in vitro analog of the pre-implantation epiblast – the founder tissue of the embryo proper. A widely held assumption, based on prior studies in the mouse system, was that naïve hPSCs are restricted in their differentiation potential toward more mature stages of epiblast development, as a prelude to gastrulation. However, over the past 5 years, a growing body of literature has demonstrated that naïve hPSCs have an expanded lineage potential toward a broad range of embryonic and extraembryonic fates and can even be used as a starting point for generating 8-cell-like cells. The most emphatic demonstration of the broad lineage potential of naïve hPSCs is their remarkable capacity to self-organize into blastocyst-like structures (“blastoids”) that model all three lineages of the pre-implantation embryo and can be cultured to post-implantation stages. Here, we discuss the broad spectrum of cell fates accessible from naïve hPSCs and the signaling pathways that guide the exit from the ground state of human pluripotency.

## Introduction

Pluripotency is a specific attribute of cells during early vertebrate development that can differentiate into all three embryonic germ layers (endoderm, mesoderm, and ectoderm) and primordial germ cells (PGCs), the progenitors of the gametes. Pluripotent cells reside transiently within the epiblast of the mammalian blastocyst and, after implantation, undergo germ layer specification during an intricate process called gastrulation. The time between implantation and gastrulation is highly compressed in the mouse (ca. 2–3 days), but extends to a period of approximately one week in primates, during which expression of pluripotency genes remains relatively stable [1]. It has been postulated that pluripotent cells transition from a pre-implantation (also known as “naïve”) epiblast identity into a post-implantation (also known as “primed”) epiblast identity, which coincides with the acquisition of lineage priming [2]. Additionally, the early post-implantation epiblast is thought to harbor pluripotent cells with an intermediate (also known as “formative”) identity, which is devoid of overt lineage priming but has acquired competence for PGC specification [3–5].

The continuum of pluripotent states described above can be captured in vitro in the form of embryonic stem cells isolated from mouse and human embryos. Mouse embryonic stem cells (mESCs) derived in the presence of MEK and GSK3 inhibitors (2i) and the cytokine leukemia inhibitory factor (LIF) display hallmarks of the naïve pluripotent state [6], while epiblast stem cells (EpiSCs) derived from early mouse embryos in the presence of FGF and Activin represent a primed post-implantation state [7, 8]. Human embryonic stem cells (hESCs) derived under classical FGF/Activin-based conditions also display features consistent with primed pluripotency, but can be reset into a naïve state by various chemical reprogramming methods, among which PXGL and 5i/L/A are the most widely adopted. PXGL includes inhibitors of MEK (*P*D0325901), tankyrase (*X*AV939), atypical PKC (*G*ö6983), and *L*IF [9, 10], while 5i/L/A contains inhibitors of MEK (PD0325901), GSK3 (IM-12), BRAF (SB590885), SRC (WH-4–023), and ROCK (Y-27632) together with LIF and Activin [11, 12]. The naïve identity of these cells is evidenced by a gene expression profile that aligns closely with the human pre-implantation epiblast [1, 13] and epigenetic features such as DNA hypomethylation and X chromosome reactivation in female cells [14–16]. The same culture conditions can also be used to isolate naïve induced pluripotent stem cells during somatic cell reprogramming [9, 17, 18]. Thus, the isolation of naïve hPSCs requires modulation of additional signaling pathways compared to the 2i/LIF cocktail in the mouse system. Moreover, recent studies have succeeded in deriving mouse and human stem cell lines with features of formative pluripotency [4, 19]. These formative stem cells show similarity to the pre-gastrulation epiblast and are directly responsive to germ cell induction.

Naïve mESCs contribute to high-grade chimeras upon injection into mouse blastocysts and are capable of efficiently differentiating into all three germ layers in vitro. However, they typically do not have the ability to contribute to extraembryonic lineages, such as the placenta or yolk sac, except upon genetic perturbation [20]. In contrast, multiple groups have reported over the past 5 years that naïve hPSCs can be used to derive a wide array of extraembryonic cell types. This includes the direct conversion of naïve hPSCs into amnion [21], hypoblast [22–24], trophoblast [25–29], and extraembryonic mesoderm [30]. This unrestricted lineage potential of naïve hPSCs has been leveraged by several groups to promote their self-organization into blastocyst-like structures (“blastoids”) that represent all three lineages of the pre-implantation blastocyst [31–35]. Naïve hPSCs have also been used as a launching pad to induce even earlier stages of human development, namely 8-cell-like cells [36]. In this Review, we discuss the diverse repertoire of cell fates accessible from naïve hPSCs and the signaling pathways that instruct their cell fate choices. We conclude with a perspective on the evolutionary and molecular underpinnings of this seemingly unrestricted lineage potential.

## The cell fates accessible from naïve hPSCs

### Post-implantation epiblast

**Fig. 1:**
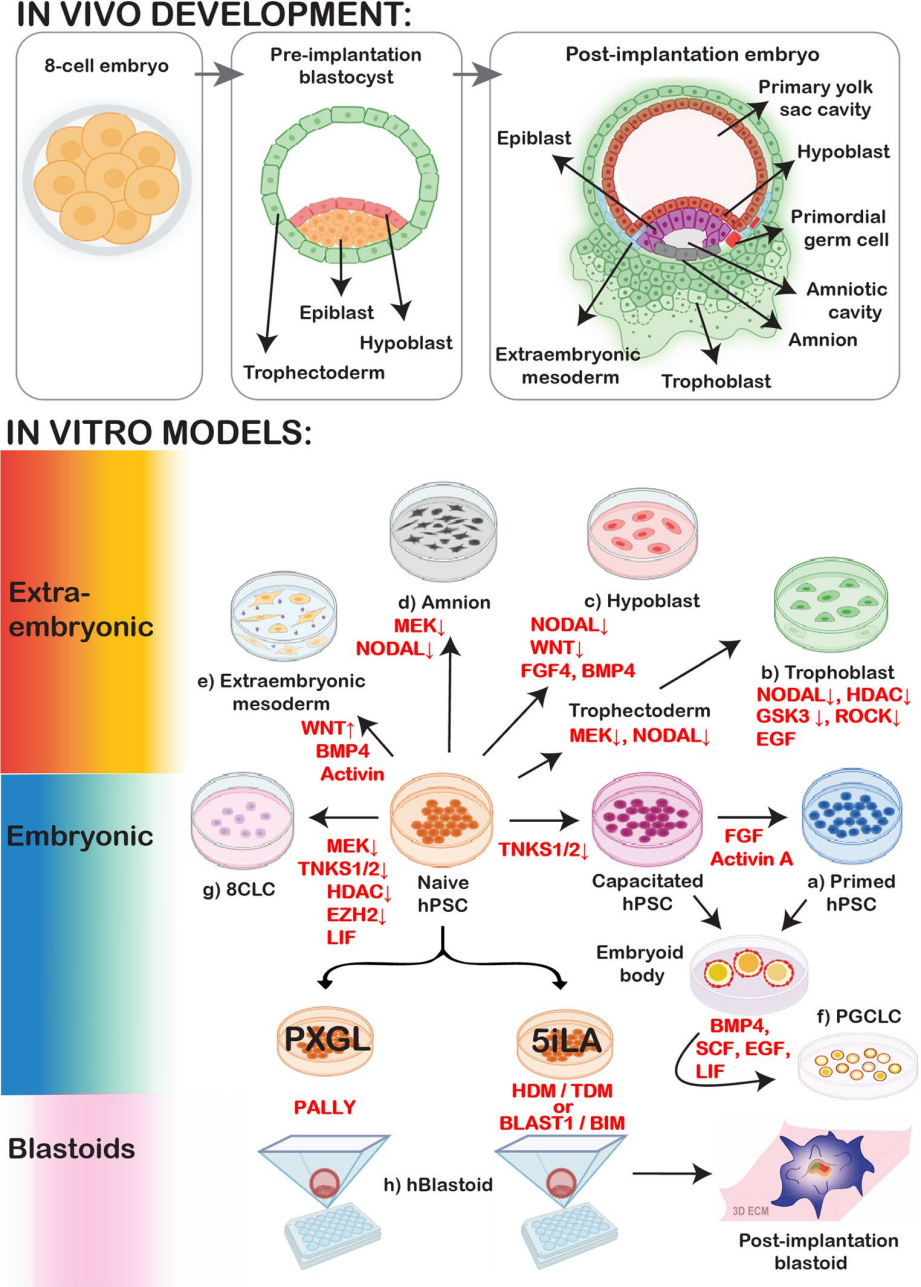
An overview of the diverse cell fates accessible from naïve hPSCs. *(Top)* Illustration of the major cell types during human embryo development spanning the 8-cell stage, pre-implantation blastocyst, and post-implantation embryo prior to gastrulation. Naïve hPSCs most closely resemble the pre-implantation epiblast. *(Bottom)* Cell states accessible from naïve hPSCs include: (**a**) Post-implantation epiblast analogs, including capacitated (also known as “formative”) hPSCs that resemble the early post-implantation epiblast and primed hPSCs that resemble the late post-implantation epiblast; (**b**) Trophoblast analogs, including a transient population of pre-implantation trophectoderm-like cells and self-renewing and bipotent hTSCs that resemble post-implantation cytotrophoblasts; (**c**) Hypoblast (also known as primitive endoderm) derivatives that contribute to yolk sac lineages; (**d**) Amnion-like cells that can be generated either from partially capacitated cells (early amnion) or fully capacitated/primed hPSCs (late amnion); (**e**) Extraembryonic mesoderm cells that are generated as a mesenchymal byproduct of hypoblast/trophoblast differentiation or more homogeneously in the presence of a GSK3 inhibitor, BMP4, and Activin; (**f**) Primordial germ cell-like cells (PGCLCs) induced from either formative or peri-gastrulation-like cells under BMP4, SCF, EGF, and LIF conditions; (**g**) 8-cell-like cells that model human ZGA and can be generated from naïve hPSCs using chemical or genetic manipulation; (**h**) Blastocyst-like structures (“blastoids”) that can be generated from naïve hPSCs derived in 5i/L/A or PXGL under different aggregation conditions

From a developmental perspective, naïve hPSCs are expected to contribute most readily to in vitro analogs of the post-implantation epiblast, the natural sequel to the pre-implantation epiblast in vivo. Indeed, naïve mESCs rapidly and homogeneously transition into a formative state upon removal of the 2i inhibitors and release in basal serum-free medium [37]. This raises the question: do naïve *human* PSCs also have the capacity to acquire a more mature epiblast identity? Several initial studies observed that naïve hPSCs lack the competence to respond to inductive cues for embryonic lineage specification, but are able to do so after re-exposure to primed culture conditions (a process known as “re-priming”) [15, 38]. Rostovskaya et al. confirmed these findings and further investigated the optimal culture conditions for endowing naïve hPSCs with the competence for germ layer induction (a process known as “capacitation”) [39]. They reported that naïve cells acquire somatic lineage competence over a period of 7 days in either E8 medium, which contains TGFβ and FGF2, or serum-free N2B27 medium. However, these media also induced significant cell death and resulted in a heterogeneous mixture of naïve and capacitated cells. In contrast, more homogeneous capacitation was observed in the presence of XAV939, a tankyrase inhibitor that degrades β-catenin and inhibits the WNT pathway (Fig. 1a). For longer term expansion of capacitated cells in vitro, XAV939 was combined with Activin A and FGF2 (XAF). Capacitated cells displayed robust proliferation over multiple passages and efficiently generated derivatives of all three germ layers, including post-mitotic neurons, definitive endoderm, and paraxial mesoderm [39].

The capacitation of naïve hPSCs offers a convenient in vitro system to investigate the molecular dynamics during the transition from naïve to formative pluripotency. Rostovskaya et al. observed two major waves of transcriptional changes. Over the first 3 days, naïve hPSCs downregulated a subset of naïve factors (*KLF4*,* TFCP2L1*), while some post-implantation markers (*TCF15*,* FGF2*, and *HES1*) were upregulated early. The second wave involved the downregulation of other naïve transcription factors, including *KLF17*,* DPPA3*, and *DPPA5*, while formative transition factors *TCF7L1* and *TCF7L2*, which are associated with canonical WNT signaling, were upregulated. Importantly, early lineage markers showed very low or undetectable expression, even at the end of the capacitation time course, suggesting that the use of XAV939 effectively shields post-implantation epiblast cells from WNT signals that induce precocious lineage priming. The close alignment between the capacitation time course and epiblast progression in cynomolgus macaque embryos indicated that this transition path models in vivo development [39].

To define epigenetic dynamics during the capacitation process, Agostinho de Sousa et al. mapped genome-wide alterations in chromatin accessibility, DNA methylation, and active and repressive histone modifications [40]. A salient observation was that capacitation is accompanied by a genome-wide increase in DNA methylation levels, while CpG islands remained low or unmethylated. Simultaneously, the repressive histone mark histone 3 lysine 27 (H3K27) trimethylation decreased between the naïve and capacitated states, but became enriched in CpG islands. Nevertheless, capacitation was unaffected by the inhibition of Polycomb Repressive Complex 2 (PRC2), which mediates H3K27 trimethylation, suggesting that capacitation is a cell-intrinsic transition independent of the global redistribution in this histone mark. This analysis also indicated that specific epigenetic modifications may play distinct roles in gene expression control between the two states: while transcription of naïve-specific genes was closely associated with H3K27 acetylation at promoters, primed-specific genes were more correlated with histone 3 lysine 4 (H3K4) trimethylation. Furthermore, long-term expansion of capacitated cells in either E8 or XAF medium resulted in an epigenetic state closely aligned with conventional primed cells in terms of chromatin accessibility, DNA methylation, and histone marks [40].

In addition to the chromatin landscape, Agostinho de Sousa et al. also investigated X chromosome inactivation (XCI) dynamics during capacitation [40]. XCI is an epigenetic phenomenon in female placental mammals that ensures adequate dosage compensation between the sexes [41, 42]. Like the cells of the pre-implantation epiblast, female naïve hPSCs have two active X chromosomes [16, 43]. Upon implantation, one X chromosome is randomly inactivated in the embryonic lineages, which is associated with the accumulation of the long non-coding RNA *XIST* and high levels of H3K27 trimethylation [44]. Consistent with their post-implantation identity, most female primed hPSCs exhibit an inactive X chromosome, but long-term culture in primed media results in the partial reactivation of the inactive X chromosome [45, 46]. This process of X chromosome erosion is associated with the loss of *XIST* expression and H3K27 trimethylation-enriched domains and presents a major impediment for the use of female hPSCs in basic and applied research [45]. Encouragingly, naïve resetting and capacitation recovered the expected characteristics of the inactive X chromosome, including *XIST* coating and accumulation of H3K27me3. This confirms a prior report by Sahakyan et al. that naïve induction followed by re-priming can reverse the eroded state of the X chromosome in long-term primed hPSC cultures [16]. Nevertheless, Agostinho de Sousa et al. also observed X chromosome erosion upon prolonged culture of capacitated hPSCs, indicating that the reinstatement of faithful XCI upon naïve reversion and capacitation is only transient. Additionally, naïve reversion does not fully recapitulate the X chromosome state of the human blastocyst (see section **i**).

### Trophoblast

The first cell fate decision in the mammalian embryo segregates the trophectoderm, the progenitor of the placenta, from the inner cell mass (ICM), which goes on to form the embryo and the yolk sac. Since the pluripotent epiblast forms within the ICM, naïve pluripotent cells are not typically expected to contribute to the trophectoderm lineage or its derivative, trophoblasts. Indeed, classical experiments demonstrated that mESCs require some form of genetic perturbation, such as overexpression of Cdx2 or downregulation of Oct4, to acquire trophectoderm fate [20]. However, several lines of evidence suggested that naïve hPSCs may have a broader lineage potential compared to their mouse counterparts. First, naïve hPSCs express several transcription factors associated with trophoblast fate, such as *ELF3*, *GCM1*, and *TFAP2C*, at elevated levels compared to their primed counterparts [14]. Second, naïve hPSCs are enriched in expression of transposon families that are associated with human cleavage embryos, most notably SVA-D and HERVK, which points to a potentially earlier developmental identity that precedes trophectoderm lineage segregation [14]. Third, naïve hPSCs share many open chromatin sites with the human placenta, highlighting an epigenetic basis for extraembryonic lineage potential [47]. These observations prompted the question of whether naïve hPSCs are capable of differentiating into human trophoblast stem cells (hTSCs), which are bipotent and self-renewing placental progenitors that were previously isolated from human embryos and first-trimester placental tissues [48]. The medium condition used to derive hTSCs includes Activin/Nodal inhibitors (*S*B431524 and *A*83-01), a histone deacetylase inhibitor (*V*alproic acid), a GSK3 inhibitor (*C*HIR99021), and a ROCK inhibitor (*Y*−27632) (“SAVECY”) [48]. Indeed, several groups reported that naïve, but not primed, hPSCs treated with this medium transition into hTSC-like cells (Fig. 1b), which could further differentiate into invasive or hormone-secreting trophoblast lineages [25–27], as well as into trophoblast organoids [49].

hTSCs share biological and transcriptional features with cytotrophoblast progenitors that arise after the embryo has undergone implantation. This raised the question whether naïve hPSCs can also give rise to trophectoderm-like cells, the precursor of the trophoblast lineage in the mammalian blastocyst. Indeed, Guo et al. and Io et al. reported that naïve hPSCs transiently acquire a trophectoderm-like state upon treatment with a MEK inhibitor and an Activin/Nodal inhibitor [28, 29]. These trophectoderm-like cells are transcriptionally aligned to human trophectoderm and can complete further differentiation into self-renewing post-implantation hTSC-like cells. Thus, the derivation of trophectoderm and hTSCs from naïve hPSCs presents an experimental paradigm to dissect the mechanisms of human trophectoderm specification, as well as an accessible source of patient-specific hTSCs to model pregnancy-associated diseases. Importantly, live-cell tracking indicated that explanted epiblast cells from human blastocysts can also differentiate into trophectoderm [28]. This suggests that the extraembryonic lineage potential of naïve hPSCs is not simply an in vitro artifact, but reflects an inherent plasticity of the naïve epiblast.

What are the molecular mechanisms that constrain or promote trophoblast differentiation from naïve hPSCs? Zijlmans et al. and Kumar et al. reported a strong enrichment of PRC2-associated H3K27me3 in the chromatin of naïve hPSCs, in particular at promoters of lineage-determining genes, including trophoblast regulators [50, 51]. These studies concluded that PRC2 activity acts as a chromatin barrier that restricts the differentiation of naive cells towards the trophoblast lineage: Zijlmans et al. reported that the inhibition of PRC2 promoted trophoblast fate induction in differentiation media, while Kumar et al. observed an increase in trophoblast and mesoderm subpopulations when PRC2 inhibition was applied under naïve culture conditions. These contrasting observations likely reflect the fact that Zijlmans et al. included XAV939 in their naïve media, which suppresses the trophectoderm program by reducing the activation of YAP [52], thereby alleviating the requirement for PRC2 to maintain naïve pluripotency. A recent study by Van Nerum et al. demonstrated that, in contrast to the repressive role of PRC2, the metabolite α-ketoglutarate promotes trophoblast differentiation in naïve hPSCs by reducing histone acetyltransferase activity and thereby weakening the pluripotency network [53]. This work identified a positive metabolic feedback loop that enhances the capacity of naïve hPSCs to specify toward trophectoderm fate.

Several groups have reported that hTSC-like cells can also be isolated directly from primed hPSCs, but this usually requires pretreatment with BMP4 or other modifications to the culture conditions [54–57]. It is important to point out that there is no evidence that post-implantation epiblast cells contribute to trophoblast development in mammalian embryos in vivo. We surmise that the direct conversion of primed hPSCs into hTSC-like cells represents a cooptation of the amniotic developmental program, which is accessible from the primed state and shares many molecular signatures with trophoblast (see section **d**). However, subtle but significant differences have been observed in hTSCs derived from primed hPSCs, such as reduced proliferation and impaired differentiation into invasive trophoblast cells, compared to hTSCs derived from naïve hPSCs [58, 59]. Work from the Arima lab suggests that the expression of the primate-specific chromosome 19 microRNA cluster in naïve, but not primed, hPSCs may be a pivotal factor: reactivation of this cluster using CRISPR epigenome editing techniques in primed hPSCs enabled the generation of hTSCs with enhanced proliferation and differentiation potential [58]. Further work will be required to determine whether additional barriers exist that limit the trophoblast potential of the primed pluripotent state.

### Hypoblast

Aside from the epiblast and the trophectoderm, the hypoblast (also known as the “primitive endoderm”) is the third major component of the blastocyst. The hypoblast arises from the ICM around 6–7 days post-fertilization, which marks the second cell lineage decision in the human embryo after the segregation of the trophectoderm [60]. The hypoblast gives rise to the yolk sac, an extraembryonic tissue that provides nutrients and oxygen to the developing embryo and serves as the initial site for blood cell production. From its beginning, the hypoblast is in constant contact with the developing embryo proper. Such proximity to the epiblast endows the hypoblast with numerous essential patterning, structural, and trophic functions [61]. Therefore, the ability to maintain hypoblast in vitro is critical to facilitate insights into human embryonic development. To date, the field has offered three approaches to derive hypoblast-like cells from naïve hPSCs.

Adhering to the same logic as for the derivation of naïve hPSCs, the first effort to isolate human hypoblast in vitro looked to the signaling requirements in the mouse system [22]. Prior studies had shown that mouse hypoblast-like cells can be derived by exposing naïve mESCs to Activin, the GSK3 inhibitor CHIR99021 (which activates WNT signaling), and LIF [62]. Building on this work, Linneberg-Agerholm et al. devised a sequential culture regime starting with *R*PMI medium supplemented with *A*ctivin, *C*HIR99021, and *L*IF (RACL) and, subsequently, N2B27 supplemented with the same components (NACL), to obtain PDGFRA-positive naïve extraembryonic endoderm (nEnd) from naïve hPSCs [22]. In contrast, treating primed hPSCs with this cocktail resulted in the generation of definitive endoderm, which can be distinguished from hypoblast by the absence of PDGFRA but presence of CXCR4. These findings suggest that the response to endoderm-inductive media in hPSCs is conditioned by the starting pluripotent state.

The concept of a conserved signaling requirement for hypoblast induction between mouse and human was challenged by other groups. Okubo et al. first applied a genetic approach to capture naive hypoblast-like cells (nHyCs) from naïve hPSCs by overexpressing the transcription factor GATA6 [63]. The resulting nHyCs expressed key markers of human hypoblast and aggregated together with naïve hPSCs to form bilaminar-disc-like structures [63]. Signaling pathway analysis of nHyCs revealed a requirement for FGF4 and BMP4 signaling during hypoblast differentiation. Surprisingly, Activin and CHIR99021 abolished the induction of PDGFRA-positive nHyCs, while an optimal efficiency of nHyC induction was attained by combining FGF4 and BMP4 with inhibitors of Activin/Nodal and WNT signaling (Fig. 1c). A similar conclusion was reached by Dattani et al., who formulated a cocktail comprising FGF2, the Activin/Nodal inhibitor A83-01, and the WNT inhibitor XAV939 (FA83X) to differentiate hypoblast from naïve hPSCs [24]. Integration with single-cell RNA-sequencing (scRNA-seq) data from human embryos indicated that, like nHyCs, FA83X cells share a strong transcriptional correlation with human blastocyst-stage hypoblast, whereas nEnd cells more closely resemble post-implantation lineages. Interestingly, this study also indicated that naive hPSC-to-hypoblast differentiation proceeds via reversion to a transitional ICM state at which the epiblast and hypoblast lineages diverge [24].

The consensus from multiple publications is that the critical function of the FGF signaling pathway in hypoblast differentiation is evolutionarily conserved from mouse to human [22, 24, 63, 64]. Two prior studies had reported that hypoblast specification in human embryos may be independent of FGF signaling [65, 66], but those experiments only applied the MEK inhibitor PD0325901 at relatively low concentrations. In contrast, Simon et al. observed a block in hypoblast differentiation in human embryos using PD0315901 at 5.0 µM [64], and Dattani et al. observed a similar phenotype by applying an upstream FGF receptor inhibitor [24]. Unlike the well-defined role of FGF signaling, the roles of the Activin/Nodal and WNT signaling pathways in hypoblast differentiation are less well conserved between mouse and human. These pathways may need to be inhibited to efficiently specify hypoblast in naïve hPSCs by suppressing trophectoderm and post-implantation epiblast fates. As we discussed previously, the WNT inhibitor XAV939 suppresses trophectoderm fate in naïve hPSCs by reducing YAP activation [52], while Activin/Nodal inhibition restricts pluripotency [67]. Further investigation is needed to elucidate the role of BMP4 in hypoblast differentiation, given that it was optional for FA83X differentiation but not for nHyC, although this observation may depend on the use of different starting naïve media. Altogether, the signaling requirements for human blastocyst-stage hypoblast have to a degree diverged from the mouse system, which further underscores the importance of a platform to study human-specific hypoblast development.

### Amnion

The amnion is a critical extraembryonic tissue that encircles the fetus during gestation. In the primate embryo, an amniotic cavity forms at the time of implantation as the amniotic epithelium becomes separated from the epiblast. In contrast, the rodent amnion is not specified until gastrulation, when it emerges by the folding of the ectoderm. Thus, in the primate embryo, the amniotic epithelium forms a physical barrier between the epiblast and trophoblast compartments, which limits the direct interactions between these tissues [68]. This sets up an important difference with the mouse embryo, where intercellular crosstalk between the epiblast and trophoblast compartments induces symmetry breaking and primitive streak formation [69]. The amnion also holds significance as the likely origin of PGCs, according to studies in non-human primate post-implantation embryos [70], and as a signaling center for primitive streak induction [71]. Therefore, the generation of a robust model of human amnion development has been of keen interest to developmental and stem cell biologists. Because the primate amnion emerges from the pre-implantation epiblast, Boroviak and Nichols postulated that the capacity to give rise to amnion may be a distinctive functional attribute of naïve hPSCs [72].

Rostovskaya et al. identified two temporally separate transcriptional trajectories of amniogenesis based on scRNA-seq data from primate embryos: an early wave of amnion specification that originates soon after implantation and shares transcriptional signatures with trophectoderm and a later wave of amnion specification that shares similarity with nonneural ectoderm [21]. By utilizing the previously mentioned capacitation model of the naïve-to-primed transition using XAV939 [39], the authors tested whether hPSCs along distinct stages of epiblast progression are competent for amniogenesis. To induce amnion fate, they combined a MEK inhibitor with an Activin/Nodal inhibitor (“PD/A83”), which simultaneously restricts the naïve-to-primed transition [73] as well as the maintenance of pluripotency [67, 74]. Rostovskaya et al. reported that the response to PD/A83 evolves as naïve hPSCs undergo capacitation: partially primed hPSCs that emerge within 3 days of capacitation model the early wave of amniogenesis, while fully primed hPSCs that emerge after stable expansion in XAF model the second wave of amniogenesis (Fig. 1d) [21]. Thus, this work validates the hypothesis from Boroviak and Nichols that naïve hPSCs provide an entry point for amniogenesis, but with the important caveat that naïve hPSCs only become competent for amniogenesis after a brief capacitation period. In fact, directly treating naïve hPSCs with the PD/A83 cocktail causes their differentiation into trophectoderm, as was also noted by Guo et al. [28].

### Extraembryonic mesoderm

The extraembryonic mesoderm (EXM) is an essential extraembryonic lineage that contributes to multiple post-implantation support structures, most notably the amnion, chorion, and yolk sac. The EXM also deposits extracellular matrix that supports the developing embryo [61]. In rodents, EXM formation occurs during gastrulation around Carnegie stage (CS) 6. In fact, the earliest population of mouse epiblast cells that migrate through the primitive streak gives rise to EXM [69]. In contrast, EXM formation can already be observed in the human CS5 peri-implantation embryo [75], long predating its formation in the post-implantation mouse embryo. This difference in developmental timing also suggests that the human and mouse EXM likely arise from drastically different signaling environments. Therefore, it is perhaps not surprising that, instead of taking a cue from culture conditions that support mouse EXM [76, 77], the first human in vitro EXM model was discovered by chance.

Pham et al. serendipitously uncovered a latent population of human EXM-like cells (EXMLC) by mapping scRNA-seq data of hTSCs derived from naïve hPSCs in SAVECY medium to an integrated dataset of human embryos [30]. These EXMLCs can be expanded for over 14 passages (~ 70 days) and exhibit expression of EXM-related markers. They can also be separated from trophoblast cells by flow sorting based on expression of the BST2 cell surface marker. In an effort to elucidate the signaling requirement for EXM differentiation and maintenance in vitro, the authors removed individual components from SAVECY medium. The combined withdrawal of the ITS-X supplement along with EGF drastically limited the EXMLC population. Because several reports have suggested a hypoblast origin of EXM [75, 78], Pham et al. also tested whether differentiation of naive hPSCs into hypoblast fate may induce EXMLCs. Remarkably, they found that most NACL cells corresponded to late EXMCs [30]. Thus, naïve hPSCs are competent to differentiate into EXMLCs as a mesenchymal byproduct in either hTSC or nEnd media.

Seeking to establish a more efficient and homogeneous method for human EXMLC induction, Niu et al. reported that naïve hPSCs treated with CHIR99021 and BMP4 (CB) rapidly differentiated into EXMLCs within four days (Fig. 1e) [79]. These EXMLCs could be stably expanded in SAVECY medium, which resulted in the upregulation of late EXM markers. Time course bulk RNA-seq analysis revealed a transient upregulation of primitive streak markers, suggesting that EXMLC differentiation from naïve hPSCs proceeds via a primitive streak-like intermediate state. In contrast, primed hPSCs treated with CB medium preferentially acquired an amnion fate. Given that Activin/Nodal signaling restricts amnion formation in primates [80], the authors supplemented CB with Activin (CBA) and found that this culture condition switches the fate of primed hPSCs from amnion to EXM [79]. Transcriptionally, EXMLCs derived from naïve hPSCs resemble pre-gastrulation EXM, while EXMLCs derived from primed hPSCs are more similar to gastrulating EXM. These data indicate that naïve and primed hPSCs can both efficiently differentiate into EXMLCs, but the initial pluripotent state influences their developmental identity.

### The germ line

The development of the germ line initiates with the specification of PGCs through a precisely regulated, stepwise process in the embryo [81]. In mice, PGCs arise from the proximal epiblast around embryonic day 6 in response to BMP signaling [82–84]. These precursors migrate through the hindgut and reach the genital ridges by E10.5–E13.5, undergoing extensive epigenetic reprogramming and sex-specific differentiation [85–87]. Male PGCs give rise to spermatogonia, which later develop into mature sperm after birth. In contrast, female PGCs differentiate into primary oocytes that remain arrested in prophase meiosis I [88–91]. Conserved between humans and mice, BMP4 also serves as a key inducer of human PGCs, which first emerge around 2–3 weeks post-fertilization [92]. Human PGCs begin migration at 4 weeks and reach the genital ridges by 5–6 weeks, where they interact with somatic cells to establish the gonads [81, 92].

The need to establish a robust method for human PGC-like cell (PGCLC) induction was one of the original motivations for deriving naïve hPSCs. Prior studies in the mouse system by Hayashi et al. demonstrated that germ cell competence is enhanced by first converting naïve mouse ESCs into epiblast-like cells (EpiLCs) in the presence of activin A and bFGF for two days [93]. These EpiLCs were subsequently differentiated into PGCLCs, a process that closely recapitulated the specification of PGCs in vivo [94]. Transplantation of PGCLCs into germ cell-deficient mice generated gametes that were capable of producing fertile offspring [93, 95, 96]. These studies raised the question of whether a similar approach would be effective at generating human PGCLCs (hPGCLCs). Indeed, Von Meyenn et al. established an hPGCLC induction system by first differentiating naïve hPSCs into human EpiLCs (hEpiLCs) for 4 days in the presence of bFGF, TGFβ, and KSR. Subsequently, the hEpiLCs were aggregated into embryoid bodies and cultured with hBMP4, hSCF, hEGF, and hLIF until day 12 to obtain hPGCLCs [87]. Yu et al. reported that self-renewing formative-like hPSCs with features of intermediate pluripotency could differentiate into hPGCLCs in response to BMP4 [19]. Thus, while naïve hPSCs appear to be refractory to direct PGCLC induction, germ cell competence is attained following a brief pulse of capacitation or using formative-like hPSCs.

Work from several labs has shown that hPGCLCs can also be induced in primed hPSCs without reversion to a capacitated or formative-like state, but upon differentiation into a germline-competent peri-gastrulation-like state. Sasaki et al. reported that transient incipient mesoderm-like cells (iMeLCs) generated from the primed state using Activin A and CHIR99021 can convert into hPGCLCs in suspension with BMP4, SCF, EGF, and LIF [97]. Similarly, Irie et al. reported that hPGCLCs can be derived efficiently from hPSCs by preconditioning the cells in a cocktail of four inhibitors (4i) that induces expression of early gastrulation-associated genes. The preconditioned hPSCs were briefly cultured in bFGF/TGFβ medium, then induced in suspension with BMP2/4, LIF, SCF, and EGF [98]. Thus, peri-gastrulation-like cells represent an alternative starting point for hPGCLC induction that bypasses the naïve state.

How do hPGCLCs generated from naïve-to-primed intermediates or peri-gastrulation-like cells compare in terms of their correspondence to PGCs in vivo and their developmental potential? Alves-Lopes et al. addressed this question by comparing hPGCLCs generated from intermediates that emerged at passage 1 of primed-to-naïve resetting (“rhPGCLCs”) to hPGCLCs generated from peri-gastrulation cells [99]. They reported that peri-gastrulation hPGCLCs express higher levels of somatic genes compared with rhPGCLCs. The delay in suppressing the somatic transcriptional program was predicted to slow down the developmental progression of peri-gastrulation PGCLCs. Indeed, rhPGCLCs exhibited accelerated upregulation of DAZL and other germline progression markers in co-culture assays with human hindgut organoids, which represent a more physiological and allogeneic environment in which to model the migration of hPGCs in vivo. Thus, the generation of hPGCLCs from naïve-to-primed intermediates opens the door to modeling hPGC specification and developing more efficient protocols for hPGCLC progression, eventually guiding them toward early-stage oogonia or pro-spermatogonia.

### 8-Cell-like cells

Recent work has demonstrated that naïve hPSCs are not only competent to differentiate into embryonic and extraembryonic lineages, but also provide a gateway to even earlier stages of human development in vitro. After fertilization, the totipotent zygote undergoes a series of cleavage divisions in which the embryo increases in cell number while maintaining its overall size. This process initially depends on a maternal store of mRNAs and proteins, but cannot be completed without zygotic genome activation (ZGA). This process occurs at the totipotent 2-cell stage in mice and at the 8-cell stage in humans [100–102]. Studying the molecular mechanisms of ZGA has been challenging due to ethical and practical restrictions on research involving human embryos. To overcome these restrictions, several research groups have established methods to derive human 8-cell-like cells (8CLCs) in vitro. Taubenschmid-Stowers et al. reported that naïve hPSCs harbor a subpopulation (~ 1.6%) of 8CLCs marked by ZGA markers, including *ZSCAN4* and *LEUTX*, and specific transposable elements, such as *HERVL* and *MLT2A1* [36]. 8CLCs show reduced expression of core pluripotency factors like SOX2 and can be identified by TPRX1 protein expression, which also marks 8-cell stage human embryos. The overexpression of the transcription factor DUX4 or spliceosome inhibition increased the fraction of 8CLCs.

As an alternative approach, Mazid et al. developed a transgene-free method for producing 8CLCs from hPSCs (Fig. 1f) [103]. They showed that a cocktail comprising small molecule inhibitors of MEK, tankyrase, histone deacetylase, and the H3K27 methyltransferase EZH2 together with LIF (4CL) induces naïve pluripotency in primed hPSCs, while further increasing the dose of histone deacetylase and EZH2 inhibition (enhanced 4CL or “e4CL”) induces 8CLCs, marked by TPRX1 [103]. By performing a small-scale chemical screen for an 8 C-specific fluorescent reporter linked to the promoter for *LEUTX*, Yu et al. discovered that the addition of a p53 activator and a PARP1 inhibitor further improved the induction of 8 C genes [104]. Thus, 8CLCs, whether induced chemically or genetically, offer an exciting in vitro model to study ZGA-like transcription and human totipotency in vitro.

### Blastoids

The discovery that naïve hPSCs have an extensive extraembryonic potential raised the question whether they may be able to self-organize into blastocyst-like structures (“blastoids”) that comprise all three lineages of the human pre-implantation embryo. Indeed, several research groups reported in quick succession that naïve hPSCs can give rise to blastoids that approximate the morphology, size, and lineage composition of human blastocysts (Fig. 1g; Table 1) [31–35]. Yu et al. established a human blastoid protocol by seeding naïve hPSCs derived in 5i/L/A in inverted-pyramidal AggreWells and sequentially treating these cell clusters with hypoblast differentiation medium (HDM) for 3 days and then trophoblast differentiation medium TDM for 4–5 days [31]. Epiblast-like cells and hypoblast-like cells emerged by day 3 in HDM, while trophoblast-like cells emerged by day 9 in TDM. However, these early blastoids still contained a substantial fraction of unclassified or unaligned cell types in integrations with human embryo reference data. Subsequent work showed that the blastoid formation rate from 5i/L/A naïve hPSCs can be increased to ~ 80–90% with few unaligned cell types by modifying the aggregation conditions (Table 1a, b) [34, 35].

**Table 1:** Blastoid induction medium formulations used by various research groups. *HDM (Hypoblast differentiation media) enhances hypoblast induction and supports better cell survival. **BLAST1 is a basal N2B27 medium used to improve blastoid formation without inhibitors for 2 days before transferring the cells to blastoid induction medium

Lab	a) Wu (2021, 2023)	b) Theunissen (2023)	c) Smith & Guo (2021)	d) Rivron (2022)
Naïve hPSCs	WIBR3, H9, YN, 46XX-hESCs, RC, YN, JB-hiPSCS, HFF hiPSCs, BJ hiPSCs	H9, H1, WIBR1, WIBR3, WIBR3 OCT4-GFP, RUES2-GLR	HNES1, niPSC HDF75, cR-ShefE	H9, ShefE, HNES1-hESCs, cR-NCRM2, niPSC 16.2.B-iPSCs
Naïve media	Modified 5i/L/A	5i/L/A	PXGL	PXGL
Blastoid induction medium				
PD0325901	+	+	+	+
A83-01	+	+	+	+
Y27632 (CEPT)	+	+	+	+
WH4-023	+	+		
IM12		+		
EGF		+		
VPA		+		
LIF	+			+
LPA	+			+
CHIR99021	+			
SB590885	+			
Activin A	+			
FGF2	+			

* Wu and Theunissen labs have developed two-step blastoid induction protocols (starting with HDM or BLAST1, respectively)

Using naïve hPSCs derived in PXGL, Yanagida et al. established an efficient method for human blastoid generation by applying a 2-day treatment with PD/A83, the same cocktail that promotes trophectoderm differentiation in naïve hPSCs [28], followed by release in basal serum-free medium alone (Table 1c) [33]. GATA3 was observed in the outer trophectoderm-like cells, while epiblast markers KLF17, NANOG, OCT4, and SOX2 were localized to the blastoid ICM by day 3. By day 4, the blastoid ICM became segregated into NANOG-positive naïve epiblast and GATA4-positive hypoblast cells. Importantly, single-cell transcriptome analysis confirmed close alignment between blastoids and human blastocysts. Also starting from naïve hPSCs derived in PXGL, Kagawa et al. established human blastoids by treating naïve cell aggregates in AggreWells with a MEK inhibitor (*P*D0325901), Activin/Nodal inhibitor (*A*83-01), Hippo inhibitor (*L*ysophosphatidic acid), LIF, and ROCK inhibitor (*Y*−27632) (PALLY) (Table 1d) [32]. This protocol enabled the development of blastoids within 4 days that closely mimicked human pre-implantation blastocysts in terms of their cellular identity and lineage composition. Additionally, these blastoids attached to a hormonally stimulated layer of endometrial epithelial cells via the polar trophectoderm, recapitulating the directionality of blastocyst attachment during human embryo implantation. Thus, naïve hPSCs not only have the ability to generate a faithful model of the pre-implantation blastocyst, but can also model the implantation process.

Do human blastoids have the ability to develop beyond the peri-implantation window? Utilizing blastoids established from 5i/L/A naïve hPSCs, Karvas et al. established a protocol for extended blastoid culture up to 21 days in vitro [34]. When cultured on appropriate three-dimensional matrices, blastoids exhibited hallmarks of early post-implantation development, including lumenogenesis within the epiblast, a bilaminar disc-like structure, and rapid expansion and diversification of trophoblast lineages. The asymmetric expression of TBXT (Brachyury) by 18 days suggested that human blastoids are capable of undergoing symmetry breaking, albeit with delayed kinetics compared to in vivo development. Another salient feature of these post-implantation blastoids was the robust presence of EXM-like cells, which invaded into placental villus structures, as observed during human placental development [105]. Integration of scRNA-seq data with a human embryo reference dataset confirmed that post-implantation blastoids harbor most of the cell types found in gastrulation-stage human embryos, although the amnion lineage was underdeveloped [34, 106]. Therefore, blastoids generated from naïve hPSCs are capable of modeling human development from pre-implantation to post-implantation stages. However, it is important to bear in mind that additional refinement will be required to enhance the spatial organization of post-implantation blastoids.

### A note on the limitations of currently available naïve hPSCs

As we have outlined above, the unbridled developmental potential of naïve hPSCs has made them an attractive platform to study early human embryogenesis. However, we must point out several important limitations that hamper the applications of currently available naïve hPSCs and the embryo models derived from them. First, current naïve hPSCs have been shown to exhibit an irreversible loss of parent-specific imprinting [14, 107]. Likely a side effect of the global hypomethylation characteristic of the naïve pluripotent state, the loss of parent-of-origin-specific epigenetic marks causes biallelic expression of imprinted genes in naïve hPSCs and their progeny. This is significant because dysregulated expression of imprinted genes has well-documented adverse effects on the developing embryo. Fischer et al. developed a biallelic reporter line targeted to the maternally imprinted *SNRPN* locus and showed that imprint erasure occurs within the first 2–3 passages of primed-to-naïve resetting [108]. They identified the KRAB zinc finger protein ZFP57 as a factor that can protect a subset of imprints when overexpressed during the initial stages of primed-to-naïve resetting. Additional work will be required to develop conditions for naïve hPSCs that support stable genomic imprinting during long-term culture.

Second, while female naïve hPSCs undergo X chromosome reactivation, their X chromosome profile does not fully resemble that of the human pre-implantation embryo. Sahakyan et al. noted that naïve hPSCs retain an epigenetic memory of the inactivated X chromosome and undergo non-random XCI upon re-priming, preferentially inactivating the X chromosome that was inactive in the primed state [16]. Excluding the cells that exhibit XCI memory, a fraction (around 20%) of female naïve hPSCs also retained two active X chromosomes after re-priming. Whether this population continues to resist XCI after more extensive culturing or differentiation still requires further elucidation. Furthermore, most naïve hPSCs exhibited monoallelic expression of the lncRNA *XIST*, which is expressed from both X chromosomes in the human blastocyst. An et al. reported that the addition of an FGF receptor inhibitor to 5i/L/A naive hPSCs enhances the fraction of naive cells with biallelic *XIST* expression and enables random XCI upon re-priming and subsequent fibroblast differentiation [109]. However, supplementing the 5i/L/A cocktail with an FGF receptor inhibitor was associated with reduced cell survival and did not support the induction of naive pluripotency. Consequently, the derivation of naïve hPSCs with biallelic *XIST* expression and competence for random XCI remains an important objective for the field.

Third, it is important to bear in mind that naïve hPSC platforms have largely been benchmarked based on their mRNA transcriptomic profile so far. However, it remains unclear whether the enhancer usage between models of the blastocyst and the actual embryo is correct. In their characterization of the transcription factor TFAP2C, Pastor et al. unveiled subtle discrepancies between the chromatin availabilities of OCT4 enhancer regions in the human blastocyst and naïve hPSCs [110]. These differences underlie a need to conduct a comparative study on the general enhancer usage pattern of key lineage-associated genes in naïve hPSCs and the human blastocyst. Furthermore, we propose that small non-coding RNA sequencing can provide an additional layer of validation to benchmark naïve hPSCs and human embryo models. A recent study by Biondic et al. demonstrated that human blastoids closely resemble human blastocysts in terms of their microRNA expression profile, but also retain expression of a subset of in-vitro-specific miRNAs that reflect their naïve stem cell origin [111]. All in all, there are still many understudied mechanisms and potential improvements to explore in naïve hPSCs. Researchers should be aware of these potential caveats before adopting these cells as a model system for early human development.

## Discussion

Initial studies on naïve human pluripotency focused on defining appropriate culture conditions for the stable induction and propagation of naïve hPSCs [112], but the past 5 years have seen significant efforts devoted toward understanding their lineage potential. As we have discussed in this Review, this work has identified a broad spectrum of embryonic and extraembryonic cell types that can be accessed from naïve hPSCs. The accumulating evidence indicates – rather paradoxically - that naïve hPSCs readily specify into extraembryonic fates, while their differentiation into embryonic lineages remains relatively inefficient, requiring a capacitation step to gain competence for embryonic germ layer or hPGCLC induction. In contrast, naïve mESCs have a more limited potential toward embryonic lineages and require genetic manipulation to contribute to extraembryonic fates [20]. This explains why naïve hPSCs self-organize into blastoids self-autonomously [31–35], while mESCs require co-aggregation with trophoblast stem cells to form blastoids [113]. Importantly, this unrestricted lineage potential is also manifest in the naïve human epiblast in vivo, which retains the ability to form trophectoderm when explanted in vitro [28]. These observations raise the question: what is so unique about the naïve state of human pluripotency in both molecular and evolutionary terms that it should exhibit such an unrestricted developmental plasticity?

Single-cell expression profiling suggested that human pre-implantation embryos undergo concurrent lineage specification and that early blastocyst cells co-express markers of all three blastocyst lineages around embryonic day 5 [43]. This would suggest the existence, albeit transiently, of a highly plastic state within the human ICM that retains potential toward embryonic and extraembryonic lineages. Additionally, recent evidence suggests that naïve hPSCs may undergo reversion into an ICM-like state before differentiating into hypoblast and trophectoderm fates [24, 114]. Regardless of the underlying molecular mechanism(s), it is evident that naïve hPSCs have an expanded developmental potential compared to their mouse counterparts. We surmise that this regulative ability may have evolved in low-fecundity mammals, such as primates, to protect the embryo against environmental insults or deleterious genetic mosaicism.

The unrestricted lineage potential of naïve hPSCs offers an unprecedented opportunity to access cell fates that cannot be easily studied in human embryos for ethical and practical reasons. We anticipate that the next decade will see continual interest in the application of naïve hPSCs to elucidate mechanisms of early lineage specification and build increasingly faithful human embryo models. Nevertheless, we must point out that important work remains to be done to improve on the genetic and epigenetic stability of naïve hPSCs, in particular as it relates to the preservation of parent-specific imprinting [108] and the induction of an epigenetic configuration of the two X chromosomes in female cells that supports biallelic *XIST* expression and random XCI as seen during human development [16, 41].

## Data Availability

Not applicable.
